# High-volume hemofiltration in adult burn patients with septic shock and acute kidney injury: a multicenter randomized controlled trial

**DOI:** 10.1186/s13054-017-1878-8

**Published:** 2017-11-25

**Authors:** Kevin K. Chung, Elsa C. Coates, David J. Smith, Rachel A. Karlnoski, William L. Hickerson, Angela L. Arnold-Ross, Michael J. Mosier, Marcia Halerz, Amy M. Sprague, Robert F. Mullins, Daniel M. Caruso, Marlene Albrecht, Brett D. Arnoldo, Agnes M. Burris, Sandra L. Taylor, Steven E. Wolf, for the Randomized controlled Evaluation of high-volume hemofiltration in adult burn patients with Septic shoCk and acUte kidnEy injury (RESCUE) Investigators, Booker T. King, Julie A. Rizzo, Jeremy C. Pamplin, Ian R. Driscoll, Evan M. Renz, Jonathan B. Lundy, Leopoldo C. Cancio, Carl W. Cruse, Christopher A. McFarren, Kimberly S. Brown, Arif Showkat, Lekha George, Aneel Kumar, Barbara Birmingham, David Hill, Mary E. Bruce, Arthur P. Sanford, David J. Leehey, Robert F. Mullins, Zaheed Hassan, Joseph R. Shaver, Bruce Friedman, Kevin N. Fosters, Michael D. Peck, Herb A. Phelan, Ramesh Saxena, James Howard, Dhaval Bhavsar, Satish Ponnuru, Michelle DeSouza, Sri G. Yarlagadda

**Affiliations:** 1 Brooke Army Medical Center, Fort Sam Houston, TX USA; 2 Uniformed Services University of the Health Sciences, Bethesda, MD USA; 3 United States Army Institute of Surgical Research, Fort Sam Houston, TX USA; 4 University of South Florida Tampa General Hospital, Tampa, FL USA; 5 University of Tennessee Firefighters’ Regional Burn Center, Memphis, TN USA; 6 Loyola University Medical Center, Maywood, IL USA; 7 Doctors Hospital Joseph M. Still Burn Center, Augusta, GA USA; 8 Arizona Burn Center Maricopa Integrated Health Systems, Phoenix, AZ USA; 9 University of Texas Southwestern Medical Center, Dallas, TX USA; 10 University of California Davis, Sacramento, CA USA

**Keywords:** High-volume hemofiltration, Burns, Septic shock, Acute kidney injury, Randomized controlled trial, Multicenter

## Abstract

**Background:**

Sepsis and septic shock occur commonly in severe burns. Acute kidney injury (AKI) is also common and often results as a consequence of sepsis. Mortality is unacceptably high in burn patients who develop AKI requiring renal replacement therapy and is presumed to be even higher when combined with septic shock. We hypothesized that high-volume hemofiltration (HVHF) as a blood purification technique would be beneficial in this population.

**Methods:**

We conducted a multicenter, prospective, randomized, controlled clinical trial to evaluate the impact of HVHF on the hemodynamic profile of burn patients with septic shock and AKI involving seven burn centers in the United States. Subjects randomized to the HVHF were prescribed a dose of 70 ml/kg/hour for 48 hours while control subjects were managed in standard fashion in accordance with local practices.

**Results:**

During a 4-year period, a total of nine subjects were enrolled for the intervention during the ramp-in phase and 28 subjects were randomized, 14 each into the control and HVHF arms respectively. The study was terminated due to slow enrollment. Ramp-in subjects were included along with those randomized in the final analysis. Our primary endpoint, the vasopressor dependency index, decreased significantly at 48 hours compared to baseline in the HVHF group (*p* = 0.007) while it remained no different in the control arm. At 14 days, the multiple organ dysfunction syndrome score decreased significantly in the HVHF group when compared to the day of treatment initiation (*p* = 0.02). No changes in inflammatory markers were detected during the 48-hour intervention period. No significant difference in survival was detected. No differences in adverse events were noted between the groups.

**Conclusions:**

HVHF was effective in reversing shock and improving organ function in burn patients with septic shock and AKI, and appears safe. Whether reversal of shock in these patients can improve survival is yet to be determined.

**Trial registration:**

Clinicaltrials.gov NCT01213914. Registered 30 September 2010.

**Electronic supplementary material:**

The online version of this article (doi:10.1186/s13054-017-1878-8) contains supplementary material, which is available to authorized users.

## Background

Severe infections resulting in septic shock occur frequently in burn patients and are associated with high mortality [1]. AKI is also a common complication in this population, with an associated mortality as high as 80% among those who need renal replacement therapy (RRT) [2]. Like other intensive care unit (ICU) populations, the cause of AKI in burns is often multifactorial. However, a major cause of AKI is the combined effect of inflammation and microcirculatory dysregulation secondary to sepsis [3, 4]. Despite advances in burn care over the past few decades, the mortality rate in burns with AKI has remained unacceptably high, especially when compared to other ICU populations [2, 5].

Recently, aggressive application of continuous venovenous hemofiltration (CVVH) was found to decrease mortality in adult patients with severe burns and AKI when compared to historical controls [6]. The greatest benefit appeared to be realized in those patients in shock at the time therapy was initiated [6]. Early data suggest that HVHF could be utilized to treat both the renal and extra-renal manifestations of AKI in the setting of septic shock [7–10]. HVHF has evolved from standard renal replacement therapies to primarily manage the metabolic consequences of AKI into one of many blood purification techniques designed to target the dysregulated immune response associated with septic shock [11–13]. Authors of a recent Cochrane meta-analysis evaluating HVHF in sepsis concluded that the data were insufficient to comment on outcomes [14]. They did, however, note no adverse effects of HVHF. The largest study included found that HVHF (70 ml/kg/hour) did not lead to a decrease in 28-day mortality or contribute to improvements in hemodynamics when compared to controls (CVVH at 35 ml/kg/hour) in a mixed ICU population [15]. No studies have included burns.

Severe burns are characterized by an augmented host response that is more pronounced than in other populations [16]. This is followed by significant catabolism with periods of major metabolic and inflammatory derangements during active infection [17]. Application of blood purification techniques in this setting may improve outcomes. We hypothesized that HVHF would improve hemodynamics in the setting of septic shock and AKI in critically ill burn patients when compared to standard care.

## Methods

After obtaining multilevel institutional review board approval, we conducted a multicenter randomized controlled trial to compare the impact of HVHF compared to controls in burn patients with septic shock and AKI. The trial was registered on ClinicalTrials.gov (NCT01213914) and was monitored by a Data Safety Monitoring Board (DSMB). Ten burn centers were selected based on the presence of an established continuous RRT capability and prior experience conducting clinical trials. The enrollment period was from February 2012 to February 2016. Centers were excluded if they were not able to enroll any study subjects within a 12-month ramp-in period.

### Subjects

We included all adults with burns who subsequently developed septic shock with AKI at least 2 days post burn. Patients with end-stage renal disease were excluded. Septic shock was defined by the American Burn Association (ABA) definition that has previously been described [18]. AKI was defined by oliguria (< 20 ml/hour) for > 24 hours or an increase in serum creatinine of > 2 mg/dl in males or > 1.5 mg/dl in females over a period of < 4 days, the same criteria utilized in a prior multicenter study [19]. Once subjects were identified as meeting the inclusion and exclusion criteria, their legally authorized representative was contacted for informed consent and enrollment within 24 hours. Once enrolled, subjects were randomized into paired groupings by age and burn size in coordination via telephone through Perry Point Cooperative Studies Center (Baltimore Research and Education Foundation, Baltimore, MD, USA).

### Intervention

Subjects randomized to the HVHF arm were initiated on CVVH at a prescribed dose of 70 ml/kg/hour for 48 hours using either the NxStage System One™ (NxStage Medical Inc., Lawrence, MA, USA) or the PRISMAFLEX System™ (Baxter Healthcare Corporation, Deerfield, IL, USA). The study dose was discontinued at 48 hours. Patients requiring RRT beyond the intervention period were prescribed the mode, dose, anticoagulation, and duration of therapy determined by the treatment team.

### Control group

Subjects randomized to the control arm underwent treatment based on local standards to include any mode of RRT, delivered at standard doses (20–35 ml/kg/hour), with the timing of treatment initiation left to be decided by the treatment team. Anticoagulation strategy was determined by the treatment team.

### Endpoints

The primary outcome measure was identified as the hemodynamics profile at 48 hours objectively measured by the vasopressor dependency index (VDI) as described previously [20] (see Additional file 1). Secondary measures included vasopressor-free days in the first 14 days, MODS score in the first 14 days [21], ICU days, and mortality. Adverse events were reported to the DSMB as directed.

### Plasma cytokine concentrations

All six cytokines (IL-6, IL-8, IL-10, IL-12, IFN-γ, and TNF-α) were measured by a sandwich ELISA method on the Theranos 3.0 device (Theranos Inc., Palo Alto, CA, USA) (see Additional file 1).

### Statistical analysis

The study was powered to detect a 4.8-unit difference in the drop of the primary endpoint from baseline at 90% power, with a type I error rate of 5% resulting in a required sample size of 120 subjects.

Continuous data are summarized as the median (25th, 75th quantile) while categorical data are summarized as proportions. Fisher’s exact test, McNemar’s test, and the Wilcoxon rank-sum test were used as appropriate. Hemodynamic parameters were compared between controls and HVHF at both hour 0 and hour 48. Median values within each group were then compared between hour 0 and hour 48 to assess the difference in the drop of the VDI from baseline. To control the type I error rate for each variable at 0.05 given four statistical tests, an alpha level of 0.0125 was used to determine significance.

Linear mixed-effect models were used to compare trends in cytokine values over the first 48 hours between the control and HVHF groups. A random intercept and slope term was included for each subject.

Data were analyzed following the intention-to-treat principle where appropriate. All tests were two-sided at a significance level of 0.05. Analyses were conducted using R Version 3.3.1 (R Core Team) or SAS/STAT software version 9.4 (SAS Institute, Inc.).

## Results

**Fig. 1:**
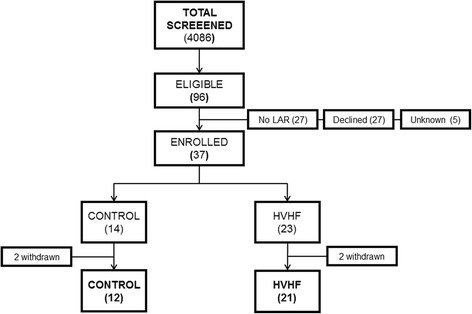
Patient flow diagram. LAR legally authorized representative, HVHF high-volume hemofiltration

Across seven participating burn centers, 4086 subjects were screened for enrollment during a 4-year enrollment period. Of those who met the inclusion criteria, a total of nine subjects were enrolled during the ramp-in phase and 28 subjects were randomized, 14 each into the control and HVHF arms. The study was terminated due to slow enrollment. Ramp-in subjects were included along with those randomized in the final analysis. Four subjects withdrew from the study. The primary endpoint was analyzed only for the subjects who remained in the study and had complete data. We applied the intent-to-treat principle for all other analyses. Figure 1 depicts the flow diagram for the trial. Baseline demographic and physiologic characteristics are presented in Table 1. Among those in the control group, all of whom were initiated on RRT upon randomization, seven and three subjects were initiated on CVVH and continuous venovenous hemodiafiltration (CVVHDF) respectively at an average delivered dose of 33 ± 3 ml/kg/hour. Two patients in the control group received intermittent hemodialysis (IHD) with a delivered clearance of 1.2 (Kt/V). All subjects in the HVHF group received CVVH at an average prescribed dose of 70 ± 1 ml/kg/hour and a delivered dose of 66 ± ml/kg/hour. In the HVHF group, 11 patients received trisodium citrate and seven patients received heparin for regional anticoagulation, while five patients received no anticoagulation. In the control group, two subjects received trisodium citrate and eight subjects received heparin for regional anticoagulation, while four subjects received no anticoagulation. Anticoagulation strategy was not found to be significantly different between the two groups (*p* = 0.11).

**Table 1:** Baseline characteristics

Variable	Control (*n* = 14)	HVHF (*n* = 23)	*p* value
Age	47 (37, 62)	50 (42, 60)	0.83
Gender (% male)	75.6	73.9	1
%TBSA	45 (29, 58)	45 (30, 60)	0.98
Inhalation (%)	36	30	1
ISS	26 (25, 47)	25 (21, 44)	0.75
ARDS (%)	57	43	0.51
MODS score	10 (9, 12)	10 (7, 14)	0.94
APACHE II score	32 (24, 35)	28 (25, 34)	0.63
MAP	70 (66, 85)	75 (64, 82)	1.00
HR	115 (101, 120)	102 (91, 116)	0.18
Hemoglobin	7.7 (7.4, 8.4)	8.2 (7.8, 9.7)	0.084
BUN	37 (32, 80)	43 (29, 50)	0.38
Creatinine	1.3 (1.1, 3.3)	2.5 (1.2, 3.1)	0.62
Lactate	1.4 (1.2, 1.6)	1.9 (1.4, 2.5)	0.22
pH	7.31 (7.25, 7.39)	7.32 (7.29, 7.38)	0.69
PFR	265 (168, 308)	211 (171, 353)	0.97
BD	–0.3 (–3.3, 1)	–2.1 (–4.3, 1.4)	0.70

Data presented as median (25th, 75th quantile) or percentage

*p* values from Wilcoxon two-sample tests for continuous variables and Fisher’s exact test for binary variables. All subjects included in summary tables via the intent-to-treat principle

*HVHF* high-volume hemofiltration, *TBSA* total body surface area, *ISS* Injury Severity Score, *ARDS* acute respiratory distress syndrome, *MODS* multiple organ dysfunction syndrome, *APACHE* Acute Physiology and Chronic Health Evaluation, *MAP* mean arterial pressure, *HR* heart rate, *BUN* blood urea nitrogen, *PFR* partial pressure of oxygen to fraction of inspired oxygen ratio, *BD* base deficit

### Primary endpoint

At the time of treatment initiation, which was a median of 2 (1, 3) hours from the time of randomization and up to 24 hours from meeting the inclusion criteria, 69% of control subjects and 50% of HVHF subjects remained on vasopressors. Our primary endpoint results (VDI) expressed as medians and quartiles are presented in Table 2 along with other hemodynamic parameters (see Additional file 1: Table S1 for all comparisons). In the HVHF group, the VDI decreased significantly at 48 hours compared to baseline (*p* = 0.007) while it remained no different in the control arm (*p* = 0.24). We also evaluated the change in the proportion of patients on vasopressors at 48 hours compared to baseline. For the control group, the percentage of patients on vasopressors did not change significantly between hour 0 and hour 48 (69% vs 50%, *p* = 0.617); while for the HVHF group, the percentage decreased significantly at hour 48 (50% vs 15%, *p* = 0.013).

**Table 2:** Hemodynamic parameters

	Control		HVHF	
Variable	Hour 0	Hour 48	Hour 0	Hour 48
MAP (mmHg)	70 (66, 85)	80 (72, 86)	75 (64, 82)	72 (68, 86)
Heart rate	115 (101, 120)	104 (97, 117)	102 (91, 116)	105 (95, 114)
Norepinephrine (μg/kg/min)	0 (0, 0.06)	0 (0, 0.06)	0 (0, 0.03)	0 (0, 0)
Vasopressin (units/hour)	0.03 (0, 0.04)	0.02 (0, 0.04)	0 (0, 0.04)	0 (0, 0)*
Modified inotropic score	4 (0, 10)	2 (0, 9.5)	1 (0, 7.13)	0 (0, 0)*
VDI	0.05 (0, 0.13)	0.02 (0, 0.12)	0.01 (0, 0.09)	0 (0, 0)*

Data presented as median (25th, 75th quantile)

*HVHF* high-volume hemofiltration, *MAP* mean arterial pressure, *VDI* vasopressor dependency index

*p < 0.0125 when comparing hour 48 to baseline (hour 0)

### Secondary endpoints

Vasopressor-free days in the first 14 days were also no different between the control and HVHF groups (3.0 (0.0, 10.3) vs 7.0 (1.0, 10.0), *p* = 0.39). At 14 days, the MODS score was no different in the control group when compared to the day of treatment initiation (10.0 (9.2, 10.5) vs 8.0 (7.0, 12.0), *p* = 0.34) while it decreased significantly in the HVHF group (10.0 (7.0, 13.5) vs 7.0 (5.0, 10.0), *p* = 0.02). Relevant end-of-study outcome measures are compared in Table 3. We did not detect any difference in ICU days or need for RRT upon hospital discharge among survivors. We also did not detect a difference in mortality at various time points.

**Table 3:** Final outcome measures

Variable	Control (*n* = 14)	HVHF (*n* = 23)	*p* value
ICU days among survivors	57 (47, 81)	67 (36, 95)	1
RRT at discharge among survivors (%)	33	38	1
14-day mortality (%)	21	17	1
28-day mortality (%)	36	22	0.45
Inhospital mortality (%)	57	65	0.73

Data presented as median (25th, 75th quantile) or percentage

All subjects included in the mortality comparison via the intent-to-treat principle

*HVHF* high-volume hemofiltration, *ICU* intensive care unit, *RRT* renal replacement therapy

### Plasma cytokines

**Fig. 2:**
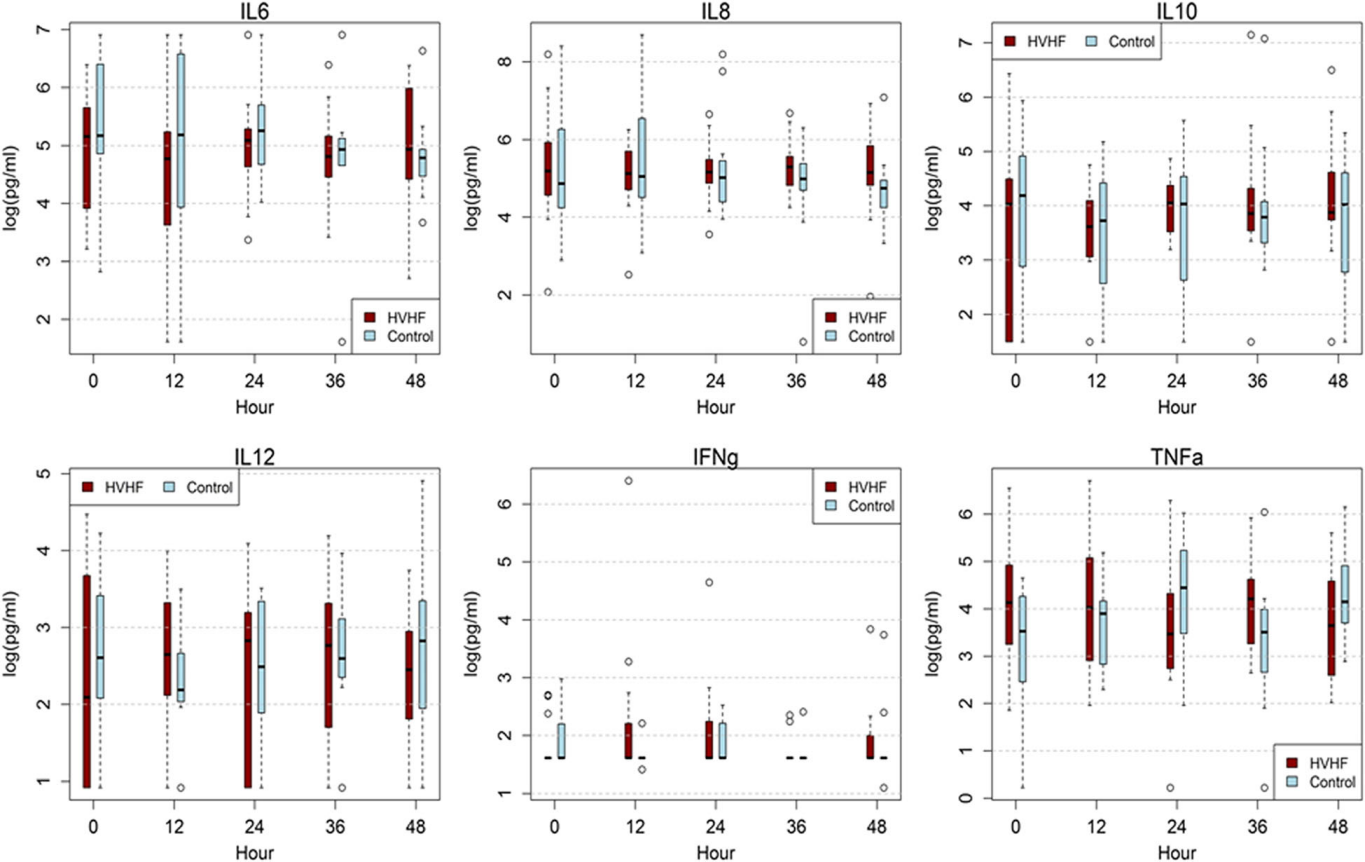
Comparison of inflammatory mediators during the 48-hour intervention. HVHF high-volume hemofiltration, IFN interferon, IL interleukin, TNF tumor necrosis factor

Of the six cytokines (IL-6, IL-8, IL-10, IL-12, IFN-γ, and TNF-α) measured over the 48-hour intervention period, none significantly decreased over time (see Fig. 2). Additionally, no cytokines were different at each time point between the two groups.

### Adverse events

No differences in adverse events were noted between the groups (see Additional file 1).

## Discussion

To date, this is the first and only controlled trial evaluating HVHF in the burn population. Despite having to stop our study early due to slow enrollment with a resultant small sample size, we detected a significant decrease in the primary endpoint over 48 hours. Thus, our data suggest that intervention with HVHF in burn patients with septic shock and concomitant AKI results in significant clinical improvement when compared to standard care. This finding was a bit unexpected and suggests that the actual effect size was greater than that assumed for our power calculation. Additionally, HVHF improved overall organ function over a 2-week period as reflected by a significant improvement of the MODS score.

While the small sample size may be a reason for restrained enthusiasm, these findings add valuable information to the limited body of literature that exists in the field of blood purification. Our detection of improved hemodynamics with HVHF in our study corroborates similar findings from previous studies. In one single-center pilot study, HVHF at 65 ml/kg/hour significantly decreased vasopressor requirements in septic shock patients with AKI [22]. In a nested cohort of 115 patients from the RENAL study, high-intensity CVVHDF, at a dose of 40 ml/kg/hour, was associated with greater improvements in MAP and vasopressor requirements when compared to controls [23]. Interestingly, the IVOIRE study, the largest trial to date to evaluate HVHF in a mixed ICU population, failed to show a benefit in hemodynamics [15]. Perhaps the difference in patient population could explain this discrepancy in our findings compared to the IVOIRE study. Burn injury is widely known to be associated with a dysregulated host response that is significant greater in magnitude and duration than any other population [17]. Regardless, it is difficult to dismiss our primary findings as being due to chance alone given the multicenter, RCT design.

The benefit of accelerating the reversal of shock is not difficult to deduce in the burn population. Among the principles of burn care is the concept of optimizing the conditions of wound healing throughout the hospitalization. The notion that shock states which impair microcirculation at the level of the wound bed will significantly impact wound healing is well accepted [24]. The extent of injured and unhealed wound burden is the greatest contributor to mortality in burns [25, 26]. Hence, any process that inhibits wound healing or results in less than optimal graft take after definitive surgery can theoretically impact overall outcomes. The study was not powered or designed to detect a difference in mortality. Given the longitudinal nature of burn care and the multiple episodes of sepsis that can occur while awaiting definitive wound coverage, it is unrealistic to expect that reversal of shock during one episode of sepsis could have an impact on inhospital mortality. Repeat treatments to reverse each episode of shock during the course of a hospitalization are likely to impact outcomes. This is particularly true if the intervention is able to preserve organ function much like HVHF did during this study.

The mechanism by which HVHF may have resulted in hemodynamic improvement in our study is somewhat unclear. Nonspecific cytokine removal in the setting of a dysregulated immune-inflammatory state is the main mechanism postulated for the improvements observed for a variety of blood purification strategies [13]. In our study, cytokine levels measured at discreet intervals for the duration of the 48-hour intervention period did not change (see Fig. 2). A recent study reported similar findings. Hemodynamic improvement with HVHF was not associated with a decrease in cytokine levels [27]. Additionally, the acid–base status was not different between the two groups (Additional file 1: Table S2). Higher intensity CRRT has been shown to improve blood pressure without any difference in acid–base status [23]. Of the many variables assessed, only BUN was different between the two groups. This suggests the possibility of better metabolic control as a reason for improvement in hemodynamics. Alternatively, a mechanism not evaluated during the course of the study, such as alteration of the neurohormonal axis or improvement of the microcirculation, may explain our findings [27–29].

In their review, Clark et al. cautioned against the routine use of HVHF for septic AKI [30]. In addition to concluding that the evidence of benefit was weak, they pointed to several concerns such as the possibility of under-dosing antibiotics and electrolyte abnormalities. It is clear that drug clearance is augmented with HVHF. However, drug pharmacokinetics is very complex and involves the consideration of multiple variables to include protein binding, the volume of distribution, and the sieving coefficient [31]. Hence, dosing should be individualized and guided by drug levels when available. Recent investigations suggest sufficient levels can be achieved by dosing to normal renal function [32–34]. Concern for therapy-related electrolyte abnormalities can easily be overcome by a standardized replacement protocol. All study sites for our trial had such protocols in place with routine monitoring and aggressive replacement. As such, no significant electrolyte abnormalities were encountered in our study, a distinct departure from what has been reported previously [15, 19].

A variety of blood purification strategies currently exist for the treatment of septic shock and can be applied in burns [13]. These include Polymyxin B-immobilized fiber therapy, plasma exchange, and hemoadsorptive columns [35–37]. Clark et al. suggest that further trials should focus on these methods in lieu of HVHF due to the resource intensiveness of the therapy [30]. However, all of these therapies harbor the same issues of insufficient clinical evidence. The most promising of these in terms of efficacy and safety, Polymyxin B hemoperfusion, is not widely available. On the other hand, HVHF is immediately available in centers that have an established continuous RRT program and does not pose nearly the same theoretical concerns that repeat treatments with plasma exchange may bring [13].

A number of limitations exist with this study. First, the study was mired by slow enrollment which resulted in a small sample size. This is despite sufficient funding and much enthusiasm within the burn community. Despite our best efforts, we fell short of our enrollment goal. Still, our findings were significant for the primary outcome and these data contribute to the body of literature that is starved of clinical data. Second, the informed consent process allowed for up to 24 hours prior to study initiation, which allowed enough time for patients to improve clinically while awaiting randomization. All of these patients received standard care which included early initiation of antibiotics and source control during the screening process. This resulted in a number of patients coming off vasopressor and correcting their laboratory abnormalities. An optimal design for this type of study would have been to alter the consent process such that subjects could be randomized as soon as they met the criteria. Our current clinical research regulatory landscape makes such study designs exceedingly difficult. This is among many key reasons why some have called for the abandonment of randomized controlled trials in the ICU [38]. Another limitation is the fact that the definition of AKI utilized for our entry criteria is now outdated. When this study design was conceived in 2009, current standardized criteria for AKI such as RIFLE (Risk, Injury, Failure, Loss, End stage), AKIN (Acute Kidney Injury Network), or KDIGO (Kidney Disease: Improving Global Outcomes) had yet to be widely adopted or their use published for burns [39]. Hence, we chose to use the AKI entry criteria utilized in the Veterans Affairs/National Institutes of Health (VA/NIH) Acute Renal Failure Trial Network paper [19]. Readers should consider this limitation when attempting to translate our findings. Finally, even within a homogeneous population such as burn patients, the phenotypic presentation of sepsis varies widely. Some patients were in profound circulatory shock with evidence of severe inflammatory dysregulation, while others had an indolent course with a low-grade level of shock. This variability makes it difficult to gauge the true impact of this therapy and may explain the lack of benefit detected in prior studies such as the IVOIRE study. Individualizing extracorporeal therapies based on the exact phenotypic presentation is the most rational approach to clinical care but is very difficult to study in large numbers. This design challenge of matching the right treatment strategy to the right patient is ongoing [40].

## Conclusion

HVHF was effective in reversing shock and improving organ function in critically ill burn patients with septic shock and AKI, and appears safe. This therapy can be considered in select patients when the right resources are available. Whether reversal of shock in these patients can improve survival is yet to be determined.

## Additional file

Additional file 1: Contains supplementary methods, **Table S1** presenting comparison of hemodynamic parameters between control and HVHF groups at baseline (hour 0) and hour 48 and comparison of change in hemodynamic parameters between baseline and hour 48 for each group, **Table S2** presenting physiologic and laboratory characteristics (mean ± SD) at hours 0, 24, and 48, **Table S3** presenting *p* values for comparisons of physiologic and laboratory characteristics of controls to HVHF subjects at 0, 24, and 48 hours, and lists IRBs. (DOCX 27 kb)

## Abbreviations

ABA: American Burn Association
AKI: Acute kidney injury
AKIN: Acute Kidney Injury Network
CVVH: Continuous venovenous hemofiltration
CVVHDF: Continuous venovenous hemodiafiltration
DSMB: Data Safety Monitoring Board
HVHF: High-volume hemofiltration
ICU: Intensive care unit
IHD: Intermittent hemodialysis
IVOIRE: hIgh VOlume in Intensive care
KDIGO: Kidney Disease: Improving Global Outcomes
MODS: Multiple organ dysfunction syndrome
RESCUE: Randomized controlled Evaluation of high-volume hemofiltration in adult burn patients with Septic shoCk and acUte kidnEy injury
RIFLE: Risk, Injury, Failure, Loss, End stage
RRT: Renal replacement therapy
VA/NIH: Veterans Affairs/National Institutes of Health
VDI: Vasopressor dependency index

## References

[1] Fitzwater J, Purdue GF, Hunt JL (2003). The risk factors and time course of sepsis and organ dysfunction after burn trauma. J Trauma.

[2] Brusselaers N, Monstrey S, Colpaert K (2010). Outcome of acute kidney injury in severe burns: a systematic review and meta-analysis. Intensive Care Med.

[3] Stewart IJ, Tilley MA, Cotant CL (2012). Association of AKI with adverse outcomes in burned military casualties. Clin J Am Soc Nephrol.

[4] Gomez H, Ince C, De Backer D (2014). A unified theory of sepsis-induced acute kidney injury: inflammation, microcirculatory dysfunction, bioenergetics, and the tubular cell adaptation to injury. Shock.

[5] Uchino S, Kellum JA, Bellomo R (2005). Acute renal failure in critically ill patients: a multinational, multicenter study. JAMA.

[6] Chung KK, Lundy JB, Matson JR (2009). Continuous venovenous hemofiltration in severely burned patients with acute kidney injury: a cohort study. Crit Care.

[7] Ronco C, Bellomo R, Homel P (2000). Effects of different doses in continuous veno-venous haemofiltration on outcomes of acute renal failure: a prospective randomised trial. Lancet.

[8] Honore PM, Jamez J, Wauthier M (2000). Prospective evaluation of short-term, high-volume isovolemic hemofiltration on the hemodynamic course and outcome in patients with intractable circulatory failure resulting from septic shock. Crit Care Med.

[9] Piccinni P, Dan M, Barbacini S (2006). Early isovolaemic haemofiltration in oliguric patients with septic shock. Intensive Care Med.

[10] Ratanarat R, Brendolan A, Ricci Z (2006). Pulse high-volume hemofiltration in critically ill patients: a new approach for patients with septic shock. Semin Dial.

[11] Cohen J (2002). The immunopathogenesis of sepsis. Nature.

[12] Azevedo LCP, Park M, Schettino GPP (2008). Novel potential therapies for septic shock. Shock.

[13] Linden K, Stewart IJ, Kreyer SF (2014). Extracorporeal blood purification in burns: a review. Burns.

[14] Borthwick EM, Hill CJ, Rabindranath KS (2017). High-volume haemofiltration for sepsis in adults. Cochrane Database Syst Rev.

[15] Joannes-Boyau O, Honore PM, Perez P (2013). High-volume versus standard-volume haemofiltration for septic shock patients with acute kidney injury (IVOIRE study): a multicenter randomized controlled trial. Intensive Care Med.

[16] Jeschke MG, Chinkes DL, Finnerty CC (2008). Pathophysiologic response to severe burn injury. Ann Surg.

[17] Seok J, Warren HS, Cuenca AG, et al. Genomic responses in mouse models poorly mimic human inflammatory diseases. Proc Natl Acad Sci U S A. 2013;110:3507–12. 10.1073/pnas.1222878110 PMC3587220 23401516

[18] Greenhalgh DG, Saffle JR, Holmes JH (2007). American Burn Association consensus conference to define sepsis and infection in burns. J Burn Care Res.

[19] Palevsky PM, Zhang JH, O’Connor TZ (2008). VA/NIH Acute Renal Failure Trial Network. Intensity of renal support in critically ill patients with acute kidney injury. N Engl J Med.

[20] Cruz DN, Antonelli M, Fumagalli R (2009). Early use of Polymyxin B Hemoperfusion in Abdominal Septic Shock: the EUPHAS Randomized Controlled Trial. JAMA.

[21] Marshall JC, Cook DJ, Christou NV (1995). Multiple organ dysfunction score: a reliable descriptor of a complex clinical outcome. Crit Care Med.

[22] Boussekey N, Chiche A, Faure K (2008). A pilot randomized study comparing high and low volume hemofiltration on vasopressor use in septic shock. Intensive Care Med.

[23] Bellomo R, Lipcsey M, Calzavacca P (2013). Early acid-base and blood pressure effects of continuous renal replacement therapy intensity in patients with metabolic acidosis. Intensive Care Med.

[24] Rowan MP, Cancio LC, Elster EA (2015). Burn wound healing and treatment: review and advancements. Crit Care.

[25] Nitzschke SL, Aden JK, Serio-Melvin ML (2014). Wound healing trajectories in burn patients and their impact on mortality. J Burn Care Res.

[26] Kagan RJ, Peck MD, Ahrenholz DH (2014). Surgical management of the burn wound and the use of skin substitutes: an expert panel white paper. J Burn Care Res.

[27] Tamme K, Maddison L, Kruusat R (2015). Effects of high volume haemodiafiltration on inflammatory response profile and microcirculation in patients with septic shock. Biomed Res Int.

[28] Khanna A, English SW, Wang XS (2017). Angiotensin II for the treatment of vasodilatory shock. N Eng J Med.

[29] Ruiz C, Hernandez G, Godoy C (2010). Sublingual microcirculatory changes during high-volume hemofiltration in hyperdynamic septic shock patients. Crit Care.

[30] Clark E, Molnar A, Joannes-Boyau O, et al. High-volume hemofiltration for septic acute kidney injury: a systematic review and meta-analysis. Crit Care 2014;18:R7. 10.1186/cc13184 PMC4057068 24398168

[31] Choi G, Gomersall CD, Tian Q (2009). Principles of antibacterial dosing in continuous renal replacement therapy. Crit Care Med.

[32] Bilgram I, Roberts JA, Wallis SC (2010). Meropenem dosing in critically ill patients with sepsis receiving high-volume continuous venovenous hemofiltration. Antimicrob Agents Chemother.

[33] Akers KS, Rowan MP, Niece KL (2015). Colistin pharmacokinetics in burn patients during continuous venovenous hemofiltration. Antimicrob Agents Chemother.

[34] Akers KS, Cota JM, Frei CR (2011). Once-daily amikacin dosing in burn patients treated with continuous venovenous hemofiltration. Antimicrob Agents Chemother.

[35] Terayama T, Yamakawa K, Umemura Y (2017). Polymyxin B hemoperfusion for sepsis and septic shock: a systematic review and meta-analysis. Surg Infect.

[36] Rimmer E, Houston BL, Kumar A (2014). The efficacy and safety of plasma exchange in patients with sepsis and septic shock: a systematic review and meta-analysis. Crit Care.

[37] Friesecke S, Stecher SS, Gross S, et al. Extracorporeal cytokine elimination as rescue therapy in refractory septic shock: a prospective single-center study. J Artif Organs. June 6 2017. Epub ahead of print. 10.1007/s10047-017-0967-4 28589286

[38] Vincent JL (2010). We should abandon randomized controlled trials in the intensive care unit. Crit Care Med.

[39] Kidney Disease: Improving Global Outcomes (KDIGO) Acute Kidney Injury Work Group (2012). KDIGO Clinical Practice Guideline for Acute Kidney Injury. Kidney Inter.

[40] Kellum JA, Gomez H, Gomez A (2015). Acute dialysis quality initiative (ADQI) XIV sepsis phenotypes and targets for blood purification in sepsis: the Bogota consensus. Shock.

