# Supplementary material for: High-volume hemofiltration in adult burn patients with septic shock and acute kidney injury: a multicenter randomized controlled trial
Source: Crit Care. 2017 Nov 25;21:289. doi: 10.1186/s13054-017-1878-8 (PMC5702112; doi:10.1186/s13054-017-1878-8)
Supplement: Additional file 1: — Contains supplementary methods, Table S1 presenting comparison of hemodynamic parameters between control and HVHF groups at baseline (hour 0) and hour 48 and comparison of change in hemodynamic parameters between baseline and hour 48 for each group, Table S2 presenting physiologic and laboratory characteristics (mean ± SD) at hours 0, 24, and 48, Table S3 presenting p values for comparisons of physiologic and laboratory characteristics of controls to HVHF subjects at 0, 24, and 48 hours, and lists IRBs. (DOCX 27 kb) [file 13054_2017_1878_MOESM1_ESM.docx]

***Vasopressor Dependency Index formula***

The VDI is calculated by combining normalized vasopressor doses at each hourly time-point into a modified inotropic score and dividing the sum by mean arterial pressure.

For example: The modified inotropic score = (norepinephrine (in mcg/kg/min)X100)+(epinephrine (in mcg/kg/min)X100)+(phenylephrine (in mcg/kg/min)X100)+(dopamine (in mcg/kg/min) X 1)+(dobutamine (in mcg/kg/min)X1)+(vasopressin (in Units/min)X100);

VDI = modified inotropic score/mean arterial pressure (in mmHg).

***Plasma cytokine measurement methodology***

Using the Theranos 3.0 device, each sample was run on two different multiplexed assay cartridge configurations customized to run three sets of the cytokine assays simultaneously. The first cartridge configuration ran IL-6, IL-8 and TNF-α assays and the second cartridge configuration ran IL-12, IL-10 and IFN-γ assays. For each of the individual sandwich ELISA assays, the capture surface was a biotin-labeled capture antibody coated on an avidin surface.

To process a sample, automated protocol executed by the device first added dilution buffer to the sample (~ 10 -fold) and then incubated this mixture on the capture surface for 10 minutes. Then an alkaline phosphatase-labeled detection antibody was incubated on the surface for 10 minutes. After the detection/antibody incubation, the surface was washed and an alkaline phosphatase chemiluminescent substrate was incubated on the surface for 10 minutes and then the resulting chemiluminescence was read. The measured luminescence was converted into a concentration based on a calibration curve for each assay. 5.7% of the samples in the HVHF arm and 4% in the control arm had concentrations above the upper limit of quantitation (ULOQ) or below the lower limit of quantitation (LLOQ). For these we set their concentration at the ULOQ or LLOQ respectively. The following chart outlines the reference standards and calibrator material utilized for each cytokine assay for the Theranos device.

| **Assay** | **Comparator Methods** | **Reference Standard / Calibrator Material** |
| --- | --- | --- |
| IL-12 | R&D Systems IL-12 Quantikine ELISA Kit | WHO IL-12 International Standard; R&D Systems IL-12 (219-IL-025) |
| IL-10 | R&D human IL-10 Immunoassay | WHO IL-10 (NIBSC code 93/722) |
| IL-8 | R&D human CXCL8/IL-8 Quantikine ELISA Kit | WHO IL-8 International Standard; R&D Systems IL-8 (208-IL-010) |
| IFNg | Abcam IFNg HS ELISA Kit | NIBSC purified IFNg Standard; R&D Systems IFNg (285-IF-100/CF) |
| IL-6 | R&D QuantiGlo human IL-6; R&D Quantikine high-sens. human IL-6 | WHO IL-6 International Standard; Apollo (Symansis) IL-6 (4006D) |
| TNFa | R&D Quantikine high-sens. human TNFa | WHO TNFa International Standard; Apollo (Symansis) TNFa (8005C) |

Table S1. Comparison of hemodynamic parameters between Control and HVHF groups at baseline (Hour 0) and Hour 48 and comparison of change in hemodynamic parameters between baseline and Hour 48 for each group.

|  | **Control vs. HVHF^a^** | | **Hour 0 vs. Hour48^b^** | |
| --- | --- | --- | --- | --- |
| **Variable** | **Hour 0** | **Hour 48** | **Control** | **HVHF** |
| MAP, mmHg^*^ | 0.573 | 0.495 | 0.083 | 0.575 |
| Heart Rate^*^ | 0.178 | 0.800 | 0.755 | 0.459 |
| Norepinephrine, mcg/kg/min^^^ | 0.915 | 0.081 | 1 | 0.035 |
| Vasopressin,  Units/hr^^^ | 0.219 | 0.009 | 0.824 | 0.018 |
| Modified Inotropic Score^^^ | 0.510 | 0.022 | 0.675 | 0.007 |
| VDI^^^ | 0.510 | 0.022 | 0.236 | 0.007 |

^a^ Wilcoxon rank sum tests, ^b^ Wilcoxon signed-rank tests

| Table S2. Physiologic and laboratory characteristics (mean ± SD) at hour 0, 24, and 48. | | | | | | |
| --- | --- | --- | --- | --- | --- | --- |
|  | **Hour 0** | | **Hour 24** | | **Hour 48** | |
| **Variable** | **Control** | **HVHF** | **Control** | **HVHF** | **Control** | **HVHF** |
| MAP | 77.36 ± 18.3 | 75.74 ± 13.9 | 79.42 ± 13.53 | 77.1 ± 11.39 | 79.75 ± 14.64 | 77.9 ± 14.68 |
| HR | 109.86 ± 13.48 | 103.7 ± 16 | 111.91 ± 19.55 | 99.9 ± 16.7 | 108.08 ± 17.78 | 104.45 ± 14.67 |
| Hb | 7.86 ± 1.26 | 8.88 ± 1.74 | 7.86 ± 1.01 | 8.82 ± 1.5 | 8.01 ± 1.09 | 8.94 ± 1.48 |
| Lactate | 1.9 ± 1.38 | 2.47 ± 2.1 | 1.62 ± 0.63 | 1.77 ± 1.04 | 1.96 ± 1.26 | 1.61 ± 0.56 |
| BUN | 55.43 ± 32.04 | 43.43 ± 20.3 | 45.18 ± 20.01 | 22.91 ± 6 | 47.03 ± 15.78 | 20.46 ± 8.4 |
| Creatinine | 2.39 ± 1.98 | 2.39 ± 1.41 | 2.14 ± 1.81 | 1.23 ± 0.73 | 1.84 ± 1.31 | 1.02 ± 0.48 |
| pH | 7.31 ± 0.11 | 7.33 ± 0.09 | 7.36 ± 0.07 | 7.38 ± 0.08 | 7.37 ± 0.09 | 7.4 ± 0.07 |
| PaO2 | 119.28 ± 34.1 | 125.59 ± 52.23 | 137.81 ± 47.75 | 108.28 ± 33.17 | 117.39 ± 37.09 | 143.97 ± 90.38 |
| FiO2 | 0.47 ± 0.08 | 0.55 ± 0.22 | 0.51 ± 0.16 | 0.45 ± 0.11 | 0.42 ± 0.1 | 0.44 ± 0.1 |
| PFR | 259.46 ± 108.33 | 267.66 ± 156.39 | 292.09 ± 112.86 | 261.05 ± 114.61 | 287.1 ± 106.47 | 355.21 ± 271.63 |
| Base Def | -1.02 ± 3.2 | -1.4 ± 3.8 | 1.07 ± 3.04 | 0.07 ± 3.34 | 0.94 ± 4.55 | 0.74 ± 2.21 |
| SBP | -^a^ | 116 ± 19 | 123.33 ± 20.79 | 119.76 ± 18.22 | 132.83 ± 18.33 | 119.4 ± 22.71 |
| WBC | 14.31 ± 8.33 | 12.85 ± 6.16 | 16.12 ± 6.13 | 15.96 ± 8.52 | 17.56 ± 8.39 | 16.79 ± 7.42 |
| HCT | 23.59 ± 3.83 | 26.8 ± 4.94 | 23.38 ± 2.85 | 26.86 ± 4.23 | 23.92 ± 3.29 | 27.05 ± 4.03 |
| Sodium | 140.07 ± 5.9 | 142.52 ± 4.86 | 140.64 ± 4.95 | 140.48 ± 4.57 | 140.18 ± 3.97 | 140 ± 3.21 |
| Potassium | 4.8 ± 1 | 4.39 ± 0.73 | 4.56 ± 0.53 | 4.25 ± 0.37 | 4.55 ± 0.65 | 4.19 ± 0.62 |
| IonizedCA | 1.07 ± 0.13 | 1.1 ± 0.1 | 1.1 ± 0.07 | 1.07 ± 0.11 | 1.07 ± 0.1 | 1.09 ± 0.12 |
| MG | 2.34 ± 0.56 | 2.16 ± 0.3 | 2.16 ± 0.41 | 2.04 ± 0.27 | 2.15 ± 0.25 | 2.07 ± 0.21 |
| PHOS | 4.62 ± 2.57 | 3.77 ± 1.06 | 4.38 ± 2.79 | 2.63 ± 0.82 | 3.65 ± 1.96 | 2.74 ± 0.92 |
| ^a^ No systolic blood pressure for control group at Hour 0 | | | | | | |

| Table S3 P-values for comparisons of physiologic and laboratory characteristics Controls to HVHF subjects at 0, 24, and 48 hours. | | | |
| --- | --- | --- | --- |
| **Variable** | **Hour0** | **Hour24** | **Hour48** |
| MAP | 1.000 | 0.736 | 0.495 |
| HR | 0.178 | 0.108 | 0.800 |
| Hb | 0.084 | 0.060 | 0.065 |
| Lactate | 0.215 | 0.895 | 0.640 |
| BUN | 0.380 | 0.000 | 0.000 |
| Creatinine | 0.616 | 0.189 | 0.116 |
| pH | 0.692 | 0.471 | 0.380 |
| PaO2 | 0.895 | 0.040 | 0.546 |
| FiO2 | 0.618 | 0.461 | 0.623 |
| PFR | 0.971 | 0.475 | 0.530 |
| Base Def | 0.695 | 0.601 | 0.668 |
| SBP | -^a^ | 0.512 | 0.106 |
| WBC | 0.541 | 0.817 | 0.935 |
| HCT | 0.056 | 0.036 | 0.051 |
| Sodium | 0.106 | 1.000 | 0.617 |
| Potassium | 0.415 | 0.110 | 0.115 |
| IonizedCA | 0.262 | 0.420 | 0.620 |
| MG | 0.430 | 0.495 | 0.395 |
| PHOS | 0.858 | 0.197 | 0.131 |
| ^a^ No systolic blood pressure for control group at Hour 0 | | | |

*Adverse events*

Throughout the study, a total of 108 adverse events (AEs) and serious adverse events (SAEs) were reported in 31 subjects. Of these 41 occurred in 12 control subjects while the rest occurred in 19 HVHF subjects. A total of 9 SAEs and 4 AEs occurred during the 48 hour intervention period, none related to treatment. Electrolyte abnormalities were reported in 4 control subjects (hyponatremia, hypernatremia, hypomagnesemia, and hypokalemia) while 2 were reported in the HVHF subjects (hypercalcemia and hyperphosphatemia). Report of secondary infection was no different as it was reported 15 times in 7 control subjects and 27 times in 11 HVHF subjects. None of the deaths were thought to be related to the intervention.

**List of Institutional Review Boards for each participating site**:

United States Army Institute of Surgical Research

- United States Army Medical Research and Materiel Command Institutional Review Board (IRB)

University of South Florida, Tampa

- University of South Florida IRB

University of Tennessee, Memphis

- University of Tennessee, Memphis IRB

Loyola University Med Center

- Loyola University Medical Center IRB

Doctors Hospital JMS Burn Center

- Western IRB

Arizona Burn Center

- Maricopa Integrated Health Systems IRB

University of Texas Southwestern

- University of Texas Southwestern IRB
